# Theory of Mind after Severe Acquired Brain Injury: Clues for Interpretation

**DOI:** 10.1155/2018/5205642

**Published:** 2018-07-05

**Authors:** U. Bivona, R. Formisano, L. Mastrilli, S. Zabberoni, C. Caltagirone, A. Costa

**Affiliations:** ^1^Fondazione Santa Lucia, IRCCS, Rome, Italy; ^2^Università telematica Niccolò Cusano, Rome, Italy; ^3^Università Tor Vergata, Rome, Italy

## Abstract

*Background. *Recently, increased interest has been shown in Theory of Mind (ToM) abilities of individuals with severe acquired brain injury (sABI). ToM impairment following sABI can be associated with altered executive functioning and/or with difficulty in decoding and elaborating emotions. Two main theoretical models have been proposed to explain the mechanisms underlying ToM in the general population:* Theory Theory* and* Simulation Theory*. This review presents and discusses the literature on ToM abilities in individuals with sABI by examining whether they sustain the applicability of the* Theory Theory* and/or* Simulation Theory* to account for ToM deficits in this clinical population. We found 32 papers that are directly aimed at investigating ToM in sABI. Results did not show the univocal predominance of one model with respect to the other in explaining ToM deficits in sABI. We hypothesised that ToM processes could be explained by coinvolvement of the two models, i.e., according to personal experience, cognitive features, or the emotional resources of the persons with sABI.

## 1. General Introduction

Theory of Mind (ToM) is the ability to infer others' intentions and beliefs [1–6]. It is crucial for sustaining social cognition, which is a prerequisite for adaptive learning and psychological satisfaction of the human being.

Two main theoretical models have been proposed to explain ToM processes. The first model is* Theory Theory* [7–12], according to which a human being infers others' intentions and beliefs by acquiring and deploying something that is quite similar to a scientific theory. Theory theorists posit that people apply general principles (e.g., rules shared within their own culture and tacitly known causal laws) regarding social relationship in order to make hypotheses about others' mental reasoning with specific involvement of executive functions, abstract reasoning, and working memory [12, 13].

In contrast to the Theory Theory, the* Simulation Theory* [10] sees ToM abilities as a result of a simulation process based on the autobiographical experience of an individual that allows “putting himself in the other's shoes”. Within this framework, the individual remodels the other's experience on himself to make predictions about the other's behaviour, and emotional-affective elaboration is supposed to play an important role. This model emerged following the discovery of mirror neurons [14, 15] which can be considered an important phylogenetic or ontogenetic precursor of the ability to assume the other's perspective. However, according to the same authors [14, 16], the mirror neuron system is not in itself able to explain the ability to ascribe propositional attitudes such as beliefs.

Several studies document that ToM may be impaired in adult individuals with psychiatric and neurological diseases (see [17] for a review); furthermore, in recent decades growing interest has been shown in investigating ToM abilities also in persons with severe acquired brain injury (sABI). Results consistently document that, compared to healthy controls, patients with sABI perform worse on ToM tasks [18–30].

## 2. ToM Functioning in Individuals with sABI

As mentioned above, ToM was found to be impaired in patients with sABI in association with altered executive functions [31–33] and/or difficulty in decoding and elaborating emotions [34–37].

It has also been recently demonstrated that ToM impairment in patients with sABI may be associated with decreased quality of life of their caregivers [29]. In this regard, note that ToM impairments can be viewed within a more general bio-psycho-social framework in which the severity of symptoms often causes poor family, social, and work re-entry outcomes.

According to the brain lesion site, dimension, and depth, patients with sABI may present with several cognitive-affective changes. Memory, attention, and executive systems are mainly involved [32, 33, 51]. Impulsivity and disinhibition are also frequently observed [37, 55, 61]. At the end of the 1970s, it was pointed out that social cognition impairment is one of the most severe behavioural consequences of an sTBI [62]. Subsequent studies confirmed that ToM can be severely affected after an sABI [18, 19, 21–24, 27–30, 63]. In particular, in individuals with severe TBI difficulty in identifying the source of an interpersonal conflict or the meaning of social behaviours has been documented [40, 64–66]. The findings of other studies were similar when patients were required to interpret nonverbal social interactions [67, 68] or to assume the perspective of a specific character in a story ([19]; see also [63], for an exhaustive review of this topic). Indeed, these patients may exhibit important changes in personality features that result in low sensitivity to others' needs [29, 69], poor interest and childishness [18, 70], and egocentrism [71].

Taken together these changes significantly increase the burden and psychophysical distress of caregivers [29, 72–74] and negatively influence patients' therapeutic outcomes [18, 73]. In fact, results of different studies document that quality of life of both patients [75] and their caregivers [29, 72–74, 76] can be significantly affected.

ToM deficits should also be viewed within the general neuropsychological profile of the patient with sABI. Indeed, in some studies [22, 33, 40, 44, 51, 65, 77], a positive correlation was found between working memory, processing speed, inhibition and the ability to be flexible, and patient's performance on ToM tasks. However, results of other studies document a possible dissociation between cognitive impairment after sABI and social cognition deficits [67, 78–80]. In this regard, some authors hypothesised that ToM and other cognitive domains should be considered as independent cognitive systems [44, 51, 54].

Particular attention should also be given to impaired self-awareness (ISA) after sABI, considered as the ability to be aware of one's own thoughts, feelings, and mental states [81], as well as “the capacity to perceive the self in relatively* objective* terms whilst maintaining a sense of* subjectivity*” [82]. Indeed, self-awareness is frequently impaired after an sABI [83–85] and is characterised by partially or totally reduced ability to recognise problems caused by damaged brain function. In a recent study of patients with sTBI and healthy controls, we demonstrated a significant relationship between ISA and perspective-taking difficulty [28], which could account for patients' difficulty in managing social relationships. In another study poor performance of patients with sTBI on ToM performance-based tasks was also found to be correlated with poor quality of life reported by their caregivers [29], which was assessed by the QOLIBRI questionnaire [86, 87]. This latter finding supports the clinical observation that caregivers may be upset by the poor sensitivity exhibited by patients with respect to the potential consequences of their behaviours (i.e., agitation, irritability, aggressiveness, apathy, and disinhibition; see [88] for more details) on the persons who care for them daily.

To summarize, available data document that ToM abilities may be reduced after sABI and that this impairment may also affect caregivers' quality of life.

## 3. Aims of the Review

Theory Theory and Simulation Theory models lead to different hypotheses about the processes implied in ToM functioning. However, the applicability and predictive value of the two models to account for ToM impairment following sABI must still be clarified.

Therefore, in this review, we present and discuss published data on ToM abilities in individuals with sABI by examining whether they sustain the applicability of the Theory Theory or Simulation Theory in accounting for ToM deficits in this clinical population.

## 4. Methods

This review was carried out using the research databases* PubMed*,* PsycINFO*, and* Scopus* to identify coherent studies on the topic investigated here up until April 2018. The literature search was completed by combining four keywords related to* brain damage* on one side and seven keywords related to* social cognition* ability on the other, as reported in Table 1. We also reviewed the reference lists of previously published reviews and all original studies to identify all relevant papers for inclusion.

All papers considered eligible for the study were then included in the review process if they met* all* of the following inclusion criteria: (1) they included patients with sABI, (2) examined ToM abilities, (3) included a healthy control group, and (4) were written in English.

After exclusion of each overlap between these three databases, only 32 papers met the above inclusion criteria. In particular, they represented 7.2% of the total number (445) of papers found eligible on PubMed, 14.5% of the total papers (220) on PsycINFO, and 9.6% of the total papers (332) on Scopus.

### 4.1. Theory Theory and sABI

As mentioned above,* Theory Theory* underlines the role of cognitive processes (i.e., logical reasoning, working memory, executive functioning, and pragmatic language) in understanding others' mental states. Therefore, it can be hypothesised that the finding of a significant association between deficits of cognitive-executive abilities and ToM impairments supports the applicability of the* Theory Theory *framework in this clinical population.

In this vein, Apperly and coll. [32] administered the false belief paradigm [27, 32, 89] to patients with sABI and found that poor ToM performance was significantly correlated with poor performance on executive tests. In patients with TBI, Henry and coll. [33] documented a significant association between performance on verbal fluency [90], i.e., a test that involves some aspects of executive functioning [91], and the Reading the Mind in the Eyes test [58]. Results of other studies that used the Faux Pas paradigm [57] seem to confirm the above association between executive and ToM functioning in individuals with sTBI [28, 51] (see Table 2).

Other investigations indirectly indicate an association between executive functioning and social cognition in sABI patients. Indeed, Channon and coll. [41] revealed a significant positive correlation between the ability to mentalize and the comprehension of sarcasm, which according to the authors could be the result of low abstract reasoning ability [41]. Moreover, by administering the* Social Problem Resolution Task *and the* Social Problem Fluency Task *[59], Channon and Crawford [40] demonstrated worse performance in patients with ABI than in healthy controls; this suggests that there may be a significant association between ToM and the ability to implement strategic social skills. More recently, McDonald et al. [49] documented a significant association between poorer social cognition and difficulty in assessing the sincerity of a speaker in patients with severe TBI.

A more specific association between working memory and ToM functioning was documented in a series of studies. Bibby and McDonald [22] administered individuals with sTBI verbal (first-order and second-order) [57] and nonverbal (cartoon) ToM tasks [22, 60] and verbal and nonverbal tasks requiring them to make general inferences. These authors found a significant association between ToM functioning and performance on tasks requiring working memory and implicit language. Honan and coll. [45] investigated whether performance on ToM tasks (i.e., different versions of a ToM procedure that varied for kind of executive demands required) depends on executive impairment in patients with severe TBI. Results were not univocal; they show that TBI patients' performance tended to be worse than that of HCs (p=0.053) in a low executive demand condition. However, the findings of this study also document a specific association between ToM performance and working memory processes (but not with flexibility and inhibition processes) as revealed by poorer ToM performance of sTBI patients than HCs in a condition with high demands on working memory processes. The association between working memory processes and ToM abilities in patients with sTBI and healthy controls was also investigated in a more recent study [56] by administering a ToM procedure (i.e., the Video Social Inference Test) in which the working memory load was manipulated. Results of this study showed that patients with sTBI performed worse than HCs on the ToM task; moreover, an association between working memory load and ToM performance was found in both experimental groups. However, by directly testing the effect of working memory load on the between group difference in ToM performance, the authors found that the reduced efficiency of sTBI patients' ToM processes could not be fully accounted for by their working memory abilities.

Taken together, these findings support the applicability of the predictions of the* Theory Theory *model to explain ToM dysfunctions in sABI because they document a significant association between performance on ToM tasks and on tests sensitive to executive functioning. However, other studies do not sustain such a relationship. In fact, Havet-Thomassin et al. [44] administered the Reading the Mind in the Eyes' test [58] and the Character Intention Task [44] to patients with sTBI and healthy controls and found no significant correlations between ToM indexes and executive performance (measured by administering the Tower of London planning task [92], the Stroop Colour Word Test [93], the Modified Card Sorting Test [94], and the Trail Making Test [95]). Similarly, other studies [27, 39, 54] found no significant association between ToM performance and various measures of executive functioning. In this vein, it should also be noted that, as discussed above, Turkstra et al. [56] showed that the ToM performance of patients with sTBI could not be fully accounted for by the involvement of working memory weakness.

In synthesis, the evidence reported above shows a heterogeneous picture that does not allow drawing firm conclusions about the involvement of executive processes in ToM functioning in patients with sABI.

### 4.2. Simulation Theory and sABI

As stated above, the* Simulation Theory *primarily posits that humans are able to “shape on the self” others' experience [14]. The ability to simulate and decode emotional experience plays a crucial role in this model with respect to pure cognitive skills. In this vein, it could be argued that emotional disturbances due to sABI are strictly related to ToM deficits in these patients. Indeed, some disturbances, such as an exaggeration or a reduction of emotional-behavioural activity, often occur after severe brain lesions. These disturbances may cause inappropriate social behaviour, impulsivity, emotional lability, irritability, loss of self-control, emotional flattening, and difficulty in recognising others' emotions and facial expressions [37, 55]. Accordingly, a significant association between emotional changes and ToM deficits could, at least theoretically, indicate the validity of the* Simulation Theory *model.

In line with this perspective, apathy was reported to be associated with poor ToM in patients with ABI [28, 42]. Moreover, Williams and Wood [36] found a significant relationship between empathic abilities (assessed by the* Balanced Emotional Empathy Scale*, BEES, [96]) and alexithymia (assessed by the* Toronto Alexithymia Scale*, TAS-20, [97]). It should also be noted that in this study more than 64% of the patients involved showed a total loss, or a significant reduction, of their empathic abilities. Moreover, Neumann and coll. [52] found that patients with moderate-to-severe TBI who showed external-oriented thinking (i.e., a tendency to avoid reasoning on (about) their own emotions) were more likely to exhibit difficulty in recognising others' emotions as well as assuming others' perspectives. De Sousa et al. [37] also documented in patients with sABI that failure to assume others' emotional perspectives was significantly associated with reduced physiological response to facial expressions with emotional valence; this suggests that there is a significant relationship between empathic abilities and capacity to code emotions (see Table 2).

In line with the above findings, in a previously mentioned study, we documented that in patients with severe TBI poor self-awareness was associated with poor performance on Faux Pas tests [28]. This finding could indicate that patients' reduced self-awareness (i.e., poor ability “to put themselves in their own shoes”) is related to their poor ability to take the other's perspective (i.e., poor ability in “putting themselves in the other's shoes”).

Taken together, the results of the above-cited studies seem to indicate that emotional processing, self-thinking, and ToM abilities are significantly interrelated; thus indirectly supports the hypotheses and predictions of the* Simulation Theory.*

However, Njomboro et al. [53] reported results that are not in line with the above conclusion, as they failed to find a significant association between affective disorders (i.e., apathy) and ToM functioning (assessed with false belief tasks) in patients with severe ABI. In particular, the results of this study document that patients with apathy do not differ from those without apathy in performing ToM tasks [53].

In summary, although in line with the Simulation Theory some findings document a significant association between ToM performance and emotional/affective variables, others (see Table 2 for details) did not show clear evidence of this association. Therefore, no firm conclusions can be made in sABI about the involvement of emotional/affective disorders in ToM functioning.

## 5. Are* Theory Theory *and* Simulation Theory* Both Involved in Explaining ToM Impairments after sABI?

The present review aimed to determine the potential applicability of both* Theory Theory* and* Simulation Theory* to account for the pattern of ToM deficits observed in the field of sABI. We do not, however, believe that the studies taken into account allow drawing firm conclusions about the superiority of one model over the other.

According to the* Theory Theory*, cognitive and, in particular, executive functions play a critical role in mediating ToM processes. Based on this assumption, we should expect to find a strong association between performance on ToM tasks and on tests investigating executive functioning in patients with sABI. Although the findings of some studies are in line with this hypothesis, other investigations failed to find an association ([22, 26, 27, 38, 43, 47, 48, 50, 54, 55]).

According to the* Simulation Theory*, instead, ToM capacities depend on the ability to simulate others' experience [10]. Therefore, the ability to code and process emotional experience by using our own mind to simulate the target's mental processes should be mainly involved in this case. Indeed, the literature examined here provides some support for the applicability of this model, in particular when low self-awareness and emotional disturbances were associated with poor ToM performance. However, also in this case, findings do not seem to be univocal [25, 26, 28, 29, 43, 46, 48, 50] (see Table 2).

The above observations could suggest that patients with sABI fail to take the other's perspective because of their reduced cognitive and/or emotional resources. Indeed, in these patients cognitive (linked to Theory Theory) and emotional/affective (linked to Simulation Theory) functions can be contemporarily or differentially involved. This evidence could support the view that the two theoretical models are not mutually exclusive in explaining ToM disorders in these individuals. Indeed, a patient with prevalent* cognitive* impairment can show ToM deficits due to difficulty in applying purely abstract logical reasoning, which hampers the ability to take into account the different elements of the presented situation. Conversely, a patient with a predominant emotional disorder can fail to take the other's perspective mainly because of difficulty implementing* simulating *mechanisms.

The evidence presented in this review does not allow documenting a clear dissociation between the Theory Theory and Simulation Theory models in sABI, but provides some clues that help explain ToM functioning also in the healthy population. In this regard, it may be useful to report the classic Tees/Crane experiment [98]. In this experiment, subjects are asked to answer a question related to the short story of Mr Crane and Mr Tees, who are scheduled to depart from the airport on different flights at the same time. Mr Crane and Mr Tees go to the airport in the same car, get caught in traffic, and arrive at the airport with a 30-minute delay with respect to their scheduled departure times. Mr Crane is told that his flight left on time and Mr Tees is told that his flight was delayed and left just five minutes before his arrival at the airport. The subjects are required to answer the question: “Who is likely to be more upset?”. The experiment showed that 96% of the subjects thought that Mr Tees would be more upset. It can be hypothesised that in order to answer the experimental questions subjects can rely on both affective (i.e., referring to the Simulation Theory) and cognitive (i.e., referring to the Theory Theory) processes. According to the Simulation Theory subjects answer* by simulating* the situation, i.e., by using their own mental state to predict how the two characters in the story feel. In fact, the subjects can take the characters' perspectives by mentally reexperiencing similar situations in their past. However, according to the Theory Theory model, different mechanisms might be involved in subjects who have never had a similar experience. In this case, individuals might apply purely abstract reasoning that would allow taking into account the different elements of the described situation.

Future studies are needed to better clarify the hypothesis of a coinvolvement of Theory Theory and Simulation Theory assumptions in explaining ToM functioning.

## 6. Sampling and Measurement 

As discussed above, we cannot draw firm conclusions about the superiority of one model with respect to the other. Indeed, some methodological limitations make it impossible to clearly understand the mechanisms underlying ToM functioning in patients with sABI.

First, the heterogeneity of samples across studies, especially those conducted on sTBI, is the first critical question. Indeed, many studies did not take into account the severity of the brain injury (i.e., mild, moderate, or severe, according to the Glasgow Coma Scale score [99]), which differentiates patients in terms of their cognitive, emotional, and behavioural functioning. Indeed, including patients with different levels of ABI severity in the same statistical analysis represents a significant bias; thus, caution is required in interpreting the results. Sample heterogeneity also makes it difficult to directly compare the results of different studies.

Another important point is the evidence that the available data are correlational in nature. This makes it difficult to examine the association between cognitive and/or emotional factors and ToM in causal terms. In this regard, no studies have investigated the possible effect on ToM ability of specific rehabilitation training for persons with sABI that is focused on the empowerment of emotional-cognitive functioning. In fact, future studies could investigate whether enhancing executive functions in patients with sABI might improve ToM in order to determine whether executive functions are involved in ToM processes in these patients.

Another critical point refers to the underestimation of poor self-awareness after sABI [28, 88, 100–102]. As stated above, individuals with reduced self-awareness can partially or totally neglect the existence of deficits related to brain injury and, consequently, they cannot accurately recognise functional consequences, including those referring to emotional, personality, and social competency changes. Moreover, it should be noted that the recovery of self-awareness is conceived as a multilevel hierarchical process [103] that starts at an* intellectual* level (patients only cognitively recognise the existence of a deficit related to brain injury) and passes through an* emergent* level (patients recognise a deficit when they are faced with it) to finally reach an* anticipatory* level of self-awareness (i.e., patients are able to plan their activities on the basis of their actual condition and residual deficits). According to this model, even when patients become intellectually self-aware of their deficits it might still be difficult for them to recognise emotional and ToM difficulties that occur in social relationships, as well as to prevent them only on the basis of their intellectual self-awareness.

The patients' difficulties in monitoring and describing their own condition can also affect their ability to reliably self-report ToM competences. Nevertheless, most of the studies that administered self-report ToM measures did not control for self-awareness disorders. Indeed, it might be paradoxical to investigate ToM problems in an individual who is unaware of the problem using explicit questions about that problem [28]. Impaired self-awareness together with cognitive deficits (mnesic and attentional in particular) could make it difficult to recall the situations cited in most self-report questionnaires (e.g., “I really get involved with the feelings of the characters in a novel” [104]) and to remember how the patient usually feels in those situations. Therefore, since individuals may provide potentially unreliable answers because of their reduced self-awareness, the selective use of self-report questionnaires to assess perspective taking in patients with sABI should be avoided [27, 28, 36, 96, 104, 105].

To surmount the above limitations, performance-based tools (see Table 3 for details) that assess patients' ability to put themselves in the shoes of the character of a story (described verbally or by pictures) and to make decisions “as if they were that character” are recommended. Indeed, these tasks require subjects to “objectively” judge what they observe during the task, not to subjectively describe their inner states. In fact, in the above-cited study [28], which investigated the possible difference between a self-report and a performance-based tool in detecting social cognition in patients with sTBI and different levels of self-awareness, it was demonstrated that only the hierarchically more complex performance-based tools (i.e., Faux Pas tests) were able to clearly discriminate between patients with poor self-awareness and patients with adequate self-awareness and healthy controls.

However, it should be noted that to date no psychometric tool has been proposed as a “gold standard” in terms of validity and reliability in assessing perspective-taking abilities after sABI. Basically, the different psychometric characteristics of the instruments utilised strongly limit the possibility of comparing results between studies. Further studies are needed to better address this issue and, in particular, to verify our suggestions regarding the usefulness of using measures to assess perspective-taking abilities in patients with sABI only after controlling for their level of self-awareness.

## 7. Conclusions

Impaired social cognition may severely affect the quality of life of persons with sABI and their caregivers [29]. Therefore, in this population understanding the mechanisms involved in ToM impairment is an important clinical issue. This review aimed to examine the applicability of* Theory Theory* and* Simulation Theory* models to explain ToM impairments in patients with sABI. These two models are based on different theoretical assumptions: according to the former, mentalizing should mainly involve cognitive strategies; conversely, the second model points out the role of emotional and simulation processes in assuming the other's perspective.

The present review highlights the difficulty of drawing firm conclusions about the applicability of the two models in the field of sABI. In fact, results from several studies show a significant association between executive functions and ToM, in line with the Theory Theory model, whereas other findings document a significant association between ToM and emotional/affective variables, congruently with the assumptions of the Simulation Theory. We interpreted the presence of studies in line with each of the proposed theoretical frameworks as potential evidence of the coinvolvement of mechanisms posited by both of these models in accounting for ToM impairments in sABI. In fact, both cognitive and emotional/affective disorders can affect the ToM abilities of these patients.

Further studies, possibly involving behavioural, neurophysiological, and neuroimaging techniques, are needed to better understand the nature of the processes underlying social cognition and, in particular, ToM functioning after sABI. Indeed, gaining knowledge about ToM functional characteristics in this clinical population could lead to the possibility of providing ad hoc rehabilitative interventions, useful not only to the patients but also to their caregiving system. In this regard, an early neuropsychological assessment of both cognitive and emotional post-sABI deficits could help rehabilitation teams treat ToM deficits better by choosing the most suitable approach based on the most available (cognitive and/or emotional) resources.

Finally, it is important that in the future researchers pay specific attention to some clinical aspects that can affect the interpretation of results. In particular, we refer to (a) the aetiology and severity of brain injury, (b) the neuropsychological and neuropsychiatric features of the patients, and (c) their level of self-awareness. All of these aspects should be taken into account in order to have homogeneous samples and more reliable findings.

## Figures and Tables

**Table 1:** Search results.

**1st keyword**	**2nd keyword**	**PubMed results**	**PsycINFO results**	**Scopus results**

Severe acquired brain injury	social cognition	188	27	34
	theory of mind	41	1	7
	mentalizing	41	13	1
	attributions	9	2	7
	perspective taking	7	4	2
	Theory Theory	135	1	26
	Simulation Theory	3	1	1

Craniocerebral trauma	social cognition	608	0	30
	theory of mind	88	1	3
	mentalizing	93	0	0
	attributions	31	0	16
	perspective taking	25	0	2
	Theory Theory	719	0	84
	Simulation Theory	15	0	10

Head injury	social cognition	633	14	217
	theory of mind	92	8	24
	mentalizing	97	1	3
	attributions	34	16	145
	perspective taking	27	5	10
	Theory Theory	815	2	433
	Simulation Theory	19	1	31

Severe traumatic brain injury	social cognition	460	117	544
	theory of mind	69	82	80
	mentalizing	69	8	7
	attributions	14	10	210
	perspective taking	16	11	16
	Theory Theory	398	12	470
	Simulation Theory	11	0	14

**Total number of items after exclusion of overlapping papers **		445	220	332

**Table 2:** Summary of studies that investigated the relationship between ToM and cognitive and emotional functions.

**Published reports **	**Characteristics of the patient group**	**Association with cognitive variables**	**Association with emotional and affective variables**
Apperly et al. [32]	12 ABI (unspecified severity) 3 HCs	Executive functions^*∗*^	Not investigated

Bara et al. [38]	13 sABI 13 HCs	Comprehension of irony, pragmatic communication (n.s.)	Not investigated

Bibby and McDonald [22]	15 sTBI 15 HCs	Working memory^*∗∗*^ (significant relationship only with non-verbal and second-order verbal ToM performance); Implicit language^*∗∗*^	Not investigated

Bivona et al. [28]	28 sTBI 28 HCs	Executive functions^*∗*^	Emotional responsiveness, Empathy (n.s.) Apathy^*∗∗*^, Self-awareness^*∗∗*^

Bivona et al. [29]	20 sTBI 20 HCs	Executive functions (n.s.)	Alexithymia (n.s.)

Bosco et al. [39]	30 moderate-to-severe TBI 30 HCs	Executive functions (n.s.)	Not investigated

Channon and Crawford [31]	7 ABI, 16 vascular, 5 cancer, 2 abscess, 1 sclerosis (unspecified severity) 60 HCs	Executive functions^*∗*^	Not investigated

Channon and Crawford [40]	10 sABI, 10 vascular (unspecified severity) 20 HCs	Comprehension of irony; Comprehension of social context and awkward situation^*∗∗*^	Not investigated

Channon et al. [41]	19 sABI 19 HCs	Comprehension of sarcasm^*∗∗*^	Not investigated

De Sousa et al. [37]	21 sTBI 22 HCs	Not investigated	Reduced psychophysiological reacti- vity during emotional stimulation^*∗∗*^

Grattan and Eslinger [42]	50 ABI, 27 infarction, 5 surgical resection of tumor, 8 aneurysm rupture, 10 closed head injury (unspecified severity)	Not investigated	Apathy^*∗∗*^

Griffin et al. [43]	11 vascular ABI (unspecified severity) 20 HCs	Executive functions, mentalistic attribution ability, humour rating (n.s.)	Emotion perception (n.s.)

Havet-Thomassin et al. [44]	17 sTBI 17 HCs	Executive functions (n.s.)	Not investigated

Henry et al. [33]	16 mild-to-moderate TBI 17 HCs	Executive functions^*∗*^	Ability to recognise facial expressions related to emotions (n.s.)

Honan et al. [45]	25 sTBI 25 HCs	High Working Memory^*∗∗*^	Not investigated

Martin and McDonald [46]	16 TBI (unspecified severity) 16 HCs	Inferential reasoning^*∗∗*^; Irony comprehension (n.s.)	Not investigated

Martin and McDonald [47]	21 ABI (unspecified severity) 21 HCs	Executive functions; Irony comprehension (n.s.)	Not investigated

McDonald and Flanagan [48]	34 sTBI 34 HCs	Detection of irony and lies (n.s.)	Ability to recognise emotional expressions of faces and voices (n.s.)

McDonald and Pearce [34]	9 ABI, 1 vascular (unspecified severity) 20 HCs	Executive functions (data applicable only to a sub-sample)^*∗*^	Ability to recognise facial expressions related to emotions^*∗*^

McDonald et al [49]	31 sTBI 25 HCs	Difficulty understanding insincere comments such as sarcasm^*∗∗*^	Emotion perception (n.s.)

Milders et al. [50]	17 moderate-to-severe TBI 17 HCs	Cognitive flexibility; Understanding Intentions and Social Situations (n.s.)	Ability to recognize emotional expressions in faces and voices (n.s.)

Milders et al. [51]	33 moderate-to-severe TBI 36 orthopedic control patients	Executive functions^*∗∗*^	Not investigated

Milders et al. [26]	33 mild-to-severe TBI 34 orthopedic control patients	Cognitive flexibility (n.s.)	Ability to recognise emotional expressions in faces and voices (n.s.)

Muller et al. [27]	15 sTBI 15 orthopedic control patients	Executive functions (n.s.)	Not investigated

Neumann and coll. [52]	60 moderate-to-severe TBI 60 orthopedic control patients	Not investigated	Alexithymia^*∗∗*^

Njomboro et al. [53]	49 ABI, 24 cerebrovascular accident, 14 head injury, 5 anoxia, 6 herpes simplex encephalitis 49 HCs	Executive functions (n.s.)	Apathy and perception of facial expressions of emotion (n. s.)

Rowe et al. [54]	31 ABI (unspecified severity) 31 HCs	Executive functions (n.s.)	Not investigated

Shamay-Tsoory et al. [35]	33 ABI, 6 vascular, 5 meningioma 18 HCs (unspecified severity)	Not investigated	Emotional variables of the stimuli^*∗∗*^

Shaw et al. [25]	19 ABI (unspecified severity) 19 HCs	Not investigated	Ability to recognise and evaluate facial expressions related to emotions (n.s.)

Spikman et al. [55]	28 moderate-to-severe ABI 33 HCs	Executive functions (n.s.)	Relationship between ToM and discrimination of emotions^*∗*^

Turkstra et al. [56]	58 moderate-to-severe TBI 66 HCs	Working Memory^*∗∗*^	Not investigated

Williams and Wood (2009)	64 sTBI 64 HCs	Not investigated	Relationship between reduced empathy and alexithymia^*∗∗*^; 64 % of TBI patients reported low emotional empathy

sABI: patients with severe acquired brain injury.

sTBI: patients with severe traumatic brain injury.

HCs: healthy control subjects.

n.s.: non-statistically significant relationship.

^*∗*^Tendency towards statistically significant relationship.

^*∗∗*^Statistically significant relationship.

**Table 3:** Main ToM tasks.

**Task**	**Authors**	**Characteristics**	**ToM aspects assessed**

First Order False Beliefs	Baron-Cohen et al. [57]	Subject has to understand one person's (the character of a short story) belief about a belief attributed to a second person.	*First level* recursive thinking (“I think that you think”); *cognitive* ToM domain.

Second Order False Beliefs	Baron-Cohen et al. [57]	Subject has to understand one person's belief about a belief attributed to a second and to a third person.	*Second level* recursive thinking (“I think that you think that he/she thinks”); *cognitive* ToM domain

Faux Pas test	Baron-Cohen et al. [57]	Subject is required to detect whether a character in a short story says something that should not be said, due to the embarrassment of the listener.	*First* and *Second level* recursive thinking; *cognitive* and *affective* ToM domains.

Reading the Mind in the Eyes test	Baron-Cohen et al. [58]	Subject is required to select the most appropriate eye-driven emotion or thought in 36 grey-scale pictures of human eyes; each of them is associated with four terms related to as many emotions.	*Affective* ToM domain.

Social Problem Resolution Task	Channon and Crawford [59]	Subjects are asked to indicate how the main character of the stories should act in several situations (responses are classified according to two criteria: social sensitivity and practical effectiveness).	*First level* recursive thinking; *cognitive* ToM domain.

Social Problem Fluency Task	Channon and Crawford [59]	Participants are asked what the main character could do in some awkward situation (i.e., theyare asked to explain why the situation might be awkward for the main character and how awkward it was).	*First level* recursive thinking; *cognitive and affective ToM domain*.

Character Intention Task	Havet-Thomassin [44]	Subjects are asked to choose, as quickly as possible, the most logical conclusion to some short comic strips (i.e., three different alternatives show a character performing an action motivated by an easily recognisable intention).	*First level* recursive thinking; *cognitive* ToM domain.

Cartoon Task	Gallagher et al. [60]; Bibby and McDonald [22]	Subjects are allowed to examine a number of ToM cartoons (based on a character's lack of knowledge about a physical situation - e.g. *the presence of a monster*) included a brief caption. The cartoons remain in front of the subjects while while they answer four questions	*First level *recursive thinking; *cognitive* ToM domain.
